# The Association between *CCL5/RANTES* SNPs and Susceptibility to HIV-1 Infection: A Meta-Analysis

**DOI:** 10.3390/v15091958

**Published:** 2023-09-20

**Authors:** Marcos Jessé Abrahão Silva, Rebecca Lobato Marinho, Pabllo Antonny Silva dos Santos, Carolynne Silva dos Santos, Layana Rufino Ribeiro, Yan Corrêa Rodrigues, Karla Valéria Batista Lima, Luana Nepomuceno Gondim Costa Lima

**Affiliations:** 1 Master Program in Epidemiology and Health Surveillance (PPGEVS), Evandro Chagas Institute (IEC), Ananindeua 67030-000, PA, Brazil; yan.13@hotmail.com; 2 Bacteriology and Mycology Section of the Evandro Chagas Institute (IEC), Ananindeua 67030-000, PA, Brazil; rebeccamarinho28@gmail.com (R.L.M.); layanarufino_ribeiro@hotmail.com (L.R.R.); karlalima@iec.gov.br (K.V.B.L.); luanalima@iec.gov.br (L.N.G.C.L.); 3 Master and PhD Program in Parasitic Biology in the Amazon (PPGBPA), Department of Natural Science (DCNA/UEPA), University of Pará State (UEPA), Belém 66087-662, PA, Brazil; antonnypabllo@gmail.com (P.A.S.d.S.); carollyne83@hotmail.com (C.S.d.S.); 4 Department of Natural Science (DCNA/UEPA), University of Pará State (UEPA), Belém 66050-540, PA, Brazil

**Keywords:** *CCL5*, genetic polymorphisms, HIV, disease association studies

## Abstract

Genetic polymorphisms in genes that encode natural ligands of CCR5 (the main human HIV coreceptor), such as *CCL5/RANTES*, can alter the levels of secretion of these peptides. This article sought to review the relationship between single nucleotide polymorphisms (SNPs) of *CCL5/RANTES* and HIV-1 disease susceptibility. A meta-analysis was conducted through 17 articles found from January 1999 to December 2022 in the PUBMED, Science Direct, Medline, and SciELO databases. A total of three SNPs were identified and investigated under their dominant genotypic model and through a fixed-effects model. In terms of the SNP rs2107538 (G > A), in Africa and Asia, it has a protective role (OR = 0.56; 95% CI = 0.41–0.76; *p* = 0.0002, and OR = 0.88; 95% CI = 0.76–1.02; *p* = 0.08, respectively). In terms of the SNP rs2280788 (C > G), in Europe and America, it shows a higher risk role (OR = 1.92; 95% CI = 1.06–3.47; *p* = 0.03, and OR = 0.94; 95% CI = 0.94–1.11; *p* = 0.04, respectively), but in the population of Asia, with its mutant allele, it has a protective role (OR = 0.76; 95% CI = 0.63–0.93; *p* = 0.007). In terms of the SNP rs2280789 (T > C), no significant associations were found. Both SNPs rs2107538 and rs2280788 have a positive transcriptional effect on the *RANTES/CCL5* gene, while SNP rs2280789 causes a decrease in gene expression levels. This study suggests that there is an association between the increased expression of *CCL5/RANTES* and a lower risk of AIDS. Therefore, further studies are needed to arrive at a definitive conclusion, and these results may help establish scientific bases for effective HIV/AIDS control strategies.

## 1. Introduction

The human immunodeficiency virus (HIV) is responsible for causing acquired immunodeficiency syndrome (AIDS), which is mainly spread through unprotected sexual intercourse, contaminated body fluids, and antiretroviral therapy (ART) failure [1]. In 2017, 36.8 million people living with HIV/AIDS (PLHIV/AIDS) were reported worldwide, including a staggering concentration of cases in eastern and southern Africa (20.6 million, ranging from 16.8 to 24.4 million), 1.94 million new cases registered, and over 40% not properly undergoing ART according to data from the Joint United Nations Program on HIV/AIDS (UNAIDS) [2]. Moreover, it seems likely that the HIV/AIDS pandemic will remain a serious public health concern due to the need for strict adherence to a lifelong combined antiretroviral medication (cART) regimen, along with long-term side effects; the rise of non-transmissible diseases as various comorbidities, including cardiovascular, liver, kidney, and neurocognitive disorders; chronic and boosted immune activation leading to inflammation that may interfere with cART-induced viral suppression; and the debate surrounding the availability of an efficient and viable treatment scheme [3,4].

Soon after HIV infection, an exacerbated drop in CD4+ T cells is often verified due to the viral cytopathic effect, such as the host’s nonspecific polyclonal activation and high viral replication; subsequently, viral replication drops to a “set point” of viremia. The AIDS stage is usually marked by a decrease in these cell counts to below 200 cells/mm^3^, which compromises the immune response against several microorganisms leading to the establishment of opportunistic infections [5]. In certain cases, patients presenting co-infections are also included in the clinical definition of AIDS, needing rapid treatment to better clinical progression [6]. To date, individuals with HIV/AIDS following proper treatment protocols have a standard treatment with cART targeting reduction and control of viral replications; this enhances life quality, although it is unable to eradicate the virus’ latent reservoir. As a result, HIV/AIDS has changed from a deadly illness to a chronic condition needing ongoing care [7].

The early viral–host interaction relies on the gp120/gp41 envelope glycoproteins (Env) recognition on the CD4 immune cells, following interaction with the C-C chemokine receptor 5 (CCR5) or C-X-C chemokine receptor type 4 (CXCR4), which function as viral co-receptors [8]. TCL-tropic strains that preferentially use CXCR4 are referred to as X4 viruses, those that are typically M-tropic use CCR5 and are referred to as R5 isolates, while dual-tropic strains preferentially use both and are referred to as R5X4 isolates. CCR5, also known as CD195, a G protein-coupled receptor (GPCR) whose production is controlled by CREB-1, is displayed on the surface of T cells, smooth muscle endothelial cells, epithelial cells, and even parenchymal cells [9].

CD8+ T cells can act in immune response against HIV with cytolytic mechanisms to suppress its transcription and are also responsible for producing soluble mediators that contribute to the antiviral response, including β−chemokines (such as CCL4/MIP-1β and CCL5/RANTES) and cytokines (such as tumor necrosis factor–alpha [TNF-α]) [10]. β-chemokines inhibit viruses from attaching to CD4 + T cells through the CCR5 co-receptor. In particular, the chemokine C-C Motif Chemokine Ligand 5 (CCL5/Regulated On Activation, Normal T-Cell Expressed and Secreted–RANTES) binds to G protein receptors and is responsible for the lymphocyte activation and cell polarization. Most cells associated with inflammation can produce CCL5/RANTES, with T cells and macrophages being the most prevalent. CCL5/RANTES can bind to CCR1, CCR3, CCR4, and CCR5, of which CCR5 has the highest affinity [11].

CCR5 has been identified as a potential target for HIV treatments, due to its relation to the receptor that enables HIV-1 infection when combined with the viral glycoprotein gp-120 [12]. For example, Maraviroc, an antiretroviral drug from the CCR5 antagonist class, joins with CCL5 to downmodulate CCR5, blocking the internalization of CCR5 and inhibiting T-cell chemotaxis [13]. In this context, genetic variants in genes that encode proteins involved in the immune response (such as *CCL5/RANTES*) can be evaluated to identify susceptibility profiles within PLHIV. The single nucleotide polymorphisms (SNPs) of *CCL5/RANTES* may alter the secretion levels of these peptides. Indeed, previous data suggested a relation between the *RANTES/CCL5* polymorphisms and the likelihood of HIV-1 infection and progression. However, the results are still conflicting or unclear [11,14,15].

Immunogenetic research allows associations based on genetic features in an individual’s genetic profile and help with the identification of specific biomarkers related to disease risk. This genetic profile of the patient helps to establish preventive measures and more appropriate pharmacological treatments for diseases, vaccine creations, and elaboration of diagnostic strategies [16]. Thus, the present study seeks to systematically review the relationship between the SNPs of *CCL5/RANTES* and HIV-1 disease susceptibility.

## 2. Material and Methods

### 2.1. Study Design and Eligibility Criteria

A systematic review and meta-analysis were performed by strategically searching the following Medical Subject Headings (MeSH) descriptors: “Chemokine CCL5 [MeSH]”, “Polymorphism, Genetic [MeSH]”, and “HIV [MeSH], with the Boolean operator “AND” on the PubMed, Medline, SciELO, and Science Direct databases. Data were collected that focused on the population, exposure, comparison, and outcome strategy (PECO), in which the population (P) was composed of PLHIV; exposure (E), as the relationship between the *CCL5/RANTES* genetic variants and HIV; comparison (C), as people presenting these genetic polymorphisms or not; and the outcome (O), as the risk of HIV-1 infection. Finally, the following guiding question was leveraged: “Which *CCL5/RANTES* genetic variations have been demonstrated to enhance HIV-1 susceptibility?”. 

The selection criteria specified that the eligible articles must be written in English, Portuguese, or Spanish, and published between January 1999 and December 2022, focused on particular types of research designs (clinical trials, case-control studies, cross-sectional studies, and cohort studies), all of which needed to be published in peer-reviewed scientific journals. The definition of HIV-1 positive cases for inclusion was guided by the individual characterizations provided in the selected studies concerning the HIV-1 positive participants within their respective cohorts [17]. A variety of publication types, including case reports, review articles, book chapters, theses, editorial guidelines, and letters to the editor, were excluded. Additionally, studies that were only accessible in abstract form, not employing a molecular biology approach to analyze the genetic variants of *RANTES/CCL5*, or that fell outside the specified time frame were deemed ineligible for inclusion.

### 2.2. Data Extraction and Methodological Quality Assessment

The identification of genetic polymorphisms in *CCL5/RANTES* was solely based on the SNPs within the timeframe for data collection encompassing all the existing literature on the subject in the databases examined. Titles and abstracts were systematically organized and sorted using Microsoft Excel software to facilitate review. To ensure the reliability of the data, two independent reviewers (MJAS and RLM) were involved in the data selection and extraction process. Should any disagreements arise concerning the inclusion and evaluation of specific studies, a third author (PASS) mediated and facilitated discussion to reach a consensus.

Data were collected in January 2023 and included the following categories: (1) authorship details, (2) year of publication, (3) methodological setting of each study, including the genotyping technique employed, (4) characteristics of the study participants, such as phenotypic definitions, geographic origins, and the source of the control group, (5) attributes of the candidate genetic variation, including mutation ID, locus, and evidence supporting its functional role, (6) outcome metrics, including the genotypes and/or allele frequencies that were most associated with the studied traits, as well as whether the study adhered to the Hardy–Weinberg equilibrium, (7) ethnic background of the study population; and (8) allele–genotypes frequencies based on the Dominant Model. This information was then consolidated into a tabular format for easier interpretation and analysis.

Quality assessment of the included studies was conducted using the standardized checklists from the Joanna Briggs Institute (JBI). Specifically, the JBI Critical Appraisal Checklist for Case-Control Studies was employed, featuring a scoring range from 0 to 10. Likewise, the JBI Checklist for Cross-Sectional Studies was used, with a scoring range from 0 to 8, as well as the JBI Checklist for Cohort Studies, which has a scoring range from 0 to 11 [18]. It is important to note that the checklist questions were scored only if the conditioning response was “Yes,” as stipulated by the guidelines [19]. This approach ensured a standardized and rigorous evaluation of the research quality across all the included studies.

### 2.3. Data Synthesis and Statistical Analysis

The process followed for the current study is presented using the PRISMA diagram, which is based on the PRISMA Statement 2020 [20]. Using the main findings from each included study’s extracted data and tabular summary, a descriptive analysis was conducted. 

Review Manager 5.4.1 (Nordic Cochrane Center, Cochrane Collaboration, Copenhagen, Sweden) was utilized to conduct the meta-analysis. For these SNPs, only genotypic analysis was carried out using a dominant genetic model. Using a fixed-effect analysis technique, the summary odds ratios (ORs) and associated 95% confidence intervals (CIs) were calculated.

According to Cochrane, the following factors are used to examine the degree of heterogeneity in a meta-analysis: between 0% and 40%, little heterogeneity; 30% to 60%, some degree of heterogeneity; and 50% to 90%, significant heterogeneity [21]. The analysis was divided into subgroups per continent. Using I^2^ statistics and the chi-square test (with a traditional threshold of significance of *p* < 0.05), the heterogeneity between papers for comparisons was evaluated [22]. To investigate the possibility of publication bias, a funnel plot was utilized.

## 3. Results

### 3.1. Literature Search

**Figure 1:**
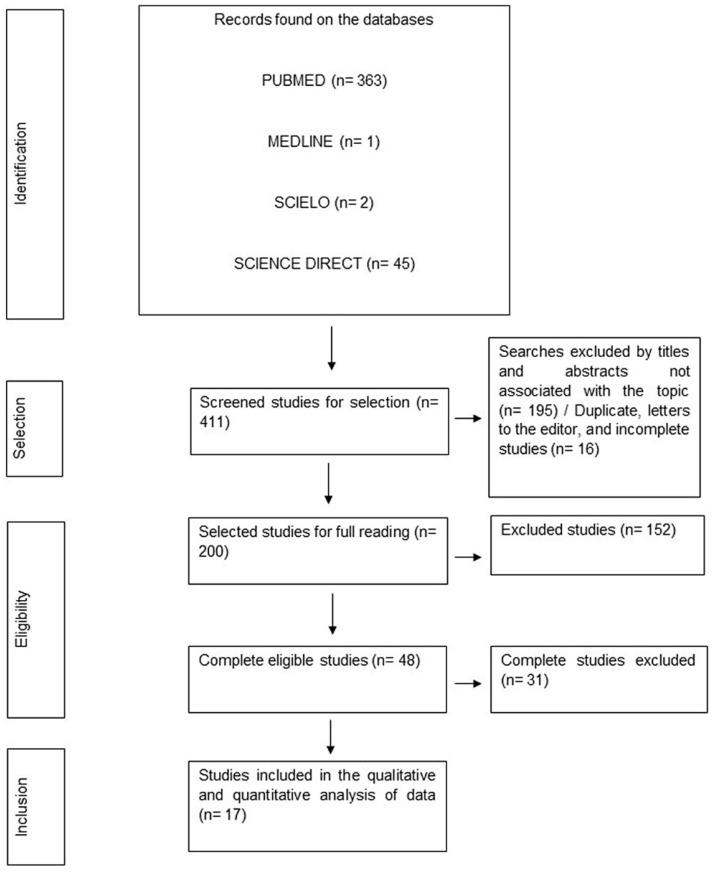
Flowchart representing the stages of selection, eligibility, and inclusion of studies for analysis. Belém, PA, Brazil (2023).

Initially, 411 articles were identified; the subsequent data selection process led to the elimination of 26 studies, comprising 5 duplicates, 5 letters to the editor, and 16 studies available only in abstract form. Additionally, 195 studies were excluded due to their irrelevance to the topic, as determined by title, abstract, and text evaluation. Upon applying the eligibility criteria and reviewing the full text of each article, the authors independently eliminated an additional 183 studies. This process resulted in the final dataset presented in this review and detailed in Figure 1.

### 3.2. Characteristics of the Articles Included in This Review

The majority of the included papers were international in scope, entirely in the English language (100%), and predominantly published by Wolters Kluwer (*n* = 10; 58.82%). Most were of the cohort study design (*n* = 14; 82.35%) and sourced from the PUBMED database (100%). Based on the Joanna Briggs Institute (JBI) scoring criteria, the methodological quality for such studies was deemed ‘high’. The analysis identified a total of three SNPs for consideration: rs2107538, rs2280788, and rs2280789.

The geographical origins of the study populations revealed a significant proportion on Asian continent, accounting for eight studies (47.06%). From a country-specific perspective, the studies were predominantly conducted in India, the USA, China, and Japan, each equally contributing with two studies each (11.76%). Furthermore, 11 (64.70%) utilized control subjects in their population-based genetic analyses. In terms of genetic polymorphism, all 17 studies conformed to the Hardy–Weinberg Equilibrium (HWE) for the three SNPs identified: rs2107538, rs2280788, and rs2280789. With respect to the association between *RANTES/CCL5* SNPs and HIV-1 infection, the most frequently examined genetic variants for susceptibility to HIV-1 were rs2107538 and rs2280788. These variants were collectively represented in 14 studies (82.35%) (Table 1).

### 3.3. Results of the Forest Plot and Publication Bias of the Meta-Analysis

**Figure 2:**
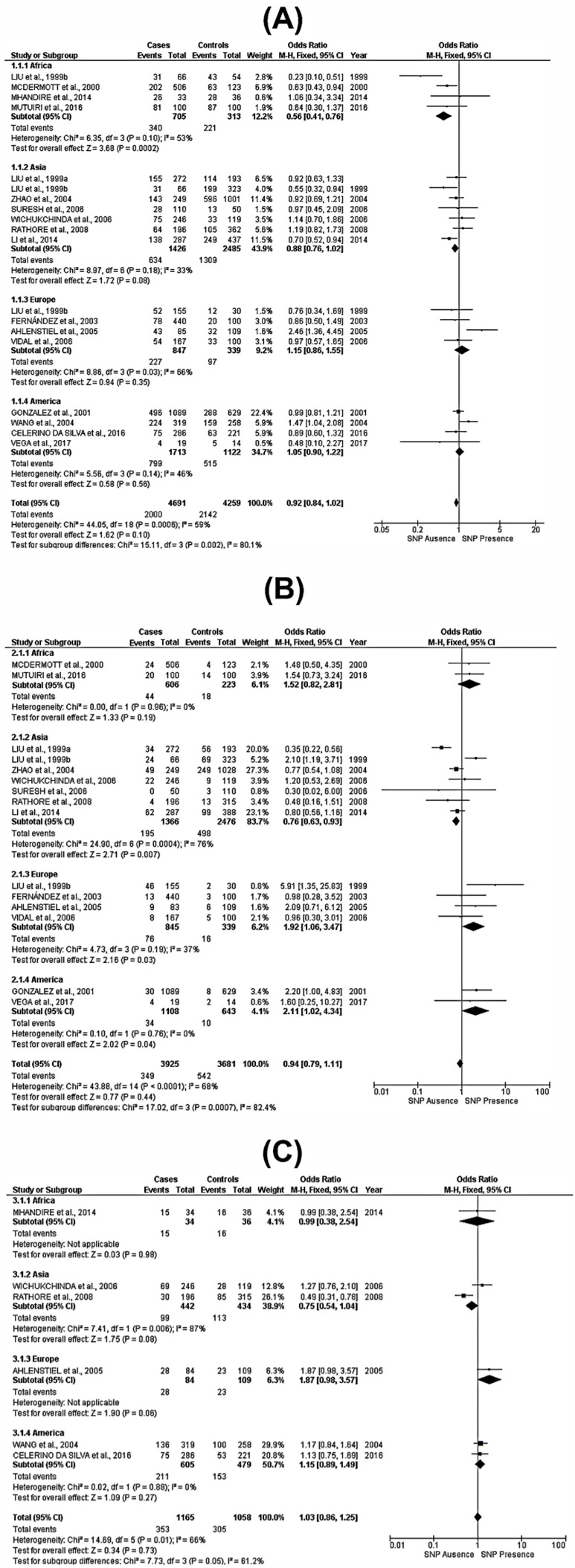
Forest plot of the associations between each SNP of the *CCL5/RANTES* gene and HIV-1 susceptibility in a dominant genotypic model. Forest plot for the SNP rs2107538, GG vs. GA + AA (**A**). Forest plot for the SNP rs2280788, CC vs. CG + GG (**B**). Forest plot for the SNP rs2280789, TT vs. TC + CC (**C**) [11,14,23,24,25,26,27,28,29,30,31,32,33,34,35,36,37].

The analysis assessed each identified SNP frequency on both cases and controls within the included studies. A geographical subgroup analysis was also performed, categorizing the data according to continents: Africa, Asia, Europe, and the Americas. Specifically, for SNP rs2107538, the analysis incorporated a total of 8950 subjects, comprising 4691 cases and 4259 controls; for SNP rs2280788, there were 3925 cases and 3681 controls; and for SNP rs2280789, there were a total of 84 cases and 109 controls (as illustrated in Figure 2).

In a comprehensive analysis, no significant global association was observed for the SNP rs2107538. However, subgroup analyses by continent revealed that both Africa and Asia demonstrated significant correlations with this SNP in terms of protective effects under a dominant genotypic model—GA + AA—(OR = 0.56; 95% CI = 0.41–0.76; *p* = 0.0002 and OR = 0.88; 95% CI = 0.76–1.02; *p* = 0.08, respectively). Populations from Europe and the Americas did not show statistical significance in this context (Figure 2A). For SNP rs2280788, the overall results also indicated no significant association with susceptibility. However, subgroup analyses revealed that populations from Europe, the Americas, and Asia exhibited statistically significant genotypes (CG+GG). Specifically, European and American populations were at a higher risk under (OR = 1.92; 95% CI = 1.06–3.47; *p* = 0.03, and OR = 0.94; 95% CI = 0.94–1.11; *p* = 0.04, respectively), whereas the Asian population displayed a protective effect (OR = 0.76; 95% CI = 0.63–0.93; *p* = 0.007) (Figure 2B). Regarding SNP rs2280789, the overall results showed no significant association for the presence of genotypes TC+ CC, as well as for the various geographical subgroups examined (Figure 2C).

**Figure 3:**
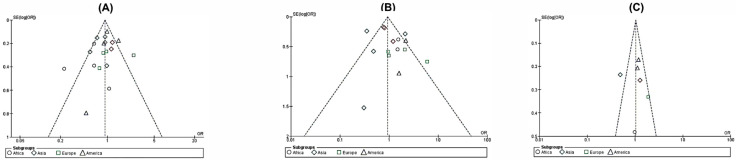
Funnel plot of each SNP of the *CCL5/RANTES* gene in this meta-analysis. Funnel plot for the analysis of the SNP rs2107538 (**A**). Funnel plot for the analysis of the SNP rs2280788 (**B**). Forest plot for the analysis of the SNP rs2280789 (**C**) [11,14,23,24,25,26,27,28,29,30,31,32,33,34,35,36,37].

In terms of subgroup analysis, no substantial heterogeneity was observed among the studies. Concerning the publication bias, the symmetrical appearance of an inverted funnel shape suggests its absence in the meta-analysis, as per the established guidelines [38]. For each individual study, the OR was plotted against the standard error of the logarithm of the OR, denoted as SE(log[OR]). The Funnel Plot graphs generated for each of the SNPs—shown in Figure 3A–C—displayed symmetry, which supports the conclusion that the meta-analysis is free from publication bias.

## 4. Discussion

### 4.1. Immune Response and AIDS

The interactions between the HIV-1 Env, the CD4 receptor, and HIV-1 coreceptors play a pivotal role in facilitating HIV-1 entry into CD4+ T cells. Cells expressing the Env glycoproteins have the capability to fuse with CD4+ target cells via an identical mechanism. This fusion process can sometimes lead to the formation of large multinucleated cells, commonly referred to as syncytia [39]. In the later stages of HIV-1 transmission, chemokine receptors CCR5 and CXCR4 often serve as the primary coreceptors for the virus, facilitating HIV-1 entry through their respective functional domains: Ba-L and JR-FL in R5-tropic HIV-1 strains and Cy in X4-tropic HIV-1 strains. CCL5/RANTES can interfere with HIV-1 cell entry and replication by competitively binding to CCR5, thereby obstructing the interaction between the HIV-1 Env gp120 and the receptor, which is essential for viral entry. Additionally, CCL5/RANTES can induce the bound receptor internalization, leading to downregulation of CCR5 on the cell surface [36,40]. It has been also established that genetic variations in the HIV-1 coreceptors (such as SNPs in *CCR5*) and in their ligands (such as SNPs in *RANTES/CCL5*) alter factors, such as infection severity [39].

Immune cells exhibiting reduced levels of CCR5, such as CD4+ T lymphocytes, tend to be more resistant to HIV-1 infection. Additionally, CD8+ T lymphocytes can inhibit the replication and pathogenesis of HIV-1 by binding to CCR5-targeting chemokines like RANTES/CCL5. This competitive binding action effectively diminishes HIV-1 entry by limiting the availability of CCR5 receptors for viral attachment. Notably, at nanomolar concentrations, RANTES/CCL5 stands out as the most potent CC-chemokine in inhibiting the replication of R5-tropic HIV-1 strains [41].

Similar to other chemokines, RANTES/CCL5 binds to one of its seven known G-protein-coupled receptors (GPCRs), which includes CCR5 at nanomolar concentrations. This binding event initiates a heterotrimeric G-protein signaling cascade. Activation of this pathway by RANTES/CCL5 through chemokine receptors leads to several intracellular changes, including transient elevations in cytosolic calcium levels (Ca2+), increases in tyrosine phosphorylation, activation of focal adhesion kinase (p125 FAK), stimulation of phospholipase D, and elevated levels of cytosolic cyclic AMP (cAMP). These molecular events collectively contribute to a range of cellular responses, including those relevant to immune function [42].

### 4.2. Host Factors and Their Relationship with the RANTES/CCL5 SNPs

The susceptibility to AIDS is likely influenced by a complex interplay of genetic, epigenetic, immunological, and environmental factors within a given population. Genetic variations like SNPs offer a lens to study these host-related aspects. Specifically, SNPs in the target genes involved in the human immune response to infection, such as *RANTES/CCL5*, can offer valuable insights, as these genetic markers have the potential to guide the development of new therapeutic strategies and predictive models, thereby providing a more targeted approach to disease management within a wide range of populations [43].

In the context of individual analyses, it was observed for SNP rs2107538 that 11 out of 16 studies addressing this variant (68.75%) reported nonsignificant associations. Similarly, for SNP rs2280788, 10 out of 15 studies (66.67%) found no statistically significant associations. For SNP rs2280789, 4 out of 10 studies (40.0%) reported nonsignificant findings. These observations highlight the contrasting data on the roles of these specific *RANTES/CCL5* SNPs across various populations. Genetic factors can vary widely among different populations due to a range of determinants, including ethnicity. This variation is often attributed to the genetic background influence, which encompasses a set of individual factors that can affect the outcome of genetic studies [44].

Candidate-gene research has identified AIDS-restriction genes, as the application of Genome Wide Association Studies (GWAS) have unveiled unique SNPs not previously associated with HIV/AIDS. These SNPs are found in a diverse array of human genes located on different chromosomes, including but not limited to *HCP5*, *HLA-C*, *CCR5*, *ZNRD1*, *DDX40*, *YPEL2*, *PRMT6*, *SOX5*, *RXRG*, *TGFBRAP1*, and *LY6*. Variant alleles in the HLA region have been highlighted by HIV/AIDS GWAS as significant predictors of HIV viral load and disease progression across various study designs and phenotypes. However, the GWAS methodology is not without limitations: due to the vast number of comparisons made and the absence of an a priori hypothesis, stringent adjustments for multiple testing are required to mitigate the risk of false-positive (type 1) errors. Unfortunately, such stringent adjustments also increase the likelihood of false-negative (type 2) errors. This poses challenges in both the identification of novel alleles and the confirmation of associations previously reported in candidate-gene studies [45]. In this sense, chemokine receptors and their ligands are playing an increasingly significant part in the illness development [33].

### 4.3. Molecular Devices Induced by the Investigated SNPs in CCL5/RANTES

The present review included three SNPs (rs2107538, rs2280788, and rs2280789) to evaluate their relationship with the *RANTES/CCL5* gene and HIV-1 infection. The SNP rs2107538 is a 2KB Upstream Variant, specifically a substitution from guanine (G) to adenine (A) in the gene promoter region. This particular SNP appears to play a significant role in the regulation of CCL5 protein synthesis. Individuals with the AA genotype show considerably higher concentrations of the CCL5 protein compared to those with the GG genotype. In essence, the presence of the A-allele mutant in the *RANTES/CCL5* rs2107538 polymorphism seems to have a positive regulatory impact on CCL5 protein production [46].

A vast number of potential cis-acting elements have been identified in the promoter region of *RANTES/CCL5*, and the production of *RANTES/CCL5* is variably controlled in different cell types [47]. According to studies, HESN patients’ CD4+ T cells generate more RANTES on average than randomly selected healthy blood donor subjects [48,49]. The SNP rs2280788 is a 2KB Upstream Variant, described for a cytosine (C) to guanine (G) substitution of nucleotide -28 (C>G) found in the gene promoter region. The -28G allele variant was found to be associated with increased levels of mRNA, causing upregulation of transcriptional activity and higher protein expression of *RANTES/CCL5* in vitro [14].

**Figure 4:**
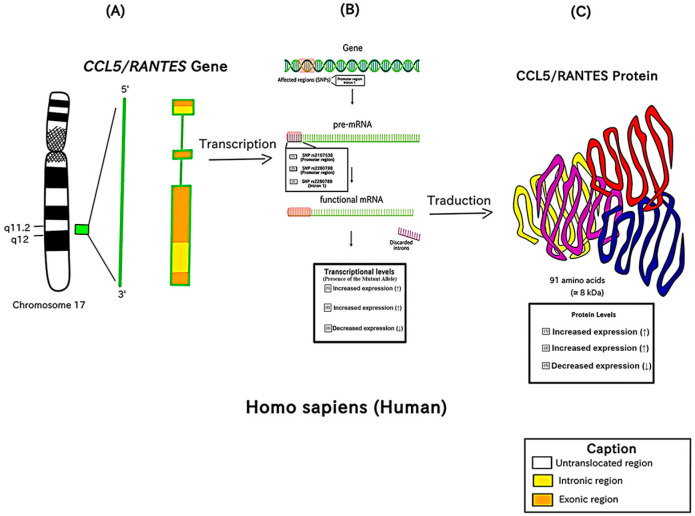
Location of the related SNPs and their association with the structure of the *CCL5/RANTES* gene and its protein. Location of the *CCL5/RANTES* gene in the human chromosome (**A**). Investigated SNPs and their effects on this gene expression (**B**). Effects of the SNPs in the protein levels (**C**).

The SNP rs2280789 (In1.1C) is a genetic variant in the intron 1 of the highlighted gene characterized by a nucleotide change from a Thymine (T) to Cytosine (C) at genetic position 35879999. An et al. (2002) [42] provided evidence for the genetic functioning of SNPs by showing that *RANTES/CCL5* rs2280789 in the promoter region predominantly controlled the transcriptional regulation of *RANTES/CCL5* and that the mutant allele C strongly decreased *RANTES/CCL5* transcriptional activity, i.e., downregulation of RANTES/CCL5. The main molecular information about these SNPs is represented in Figure 4.

### 4.4. Final Considerations and Future Perspectives

Prior meta-analyses have explored the associations between the same SNPs in the *RANTES/CCL5* gene and susceptibility to HIV-1 infection contexts. For instance, Zhao et al. (2016) employed different methodologies and covered a broader timeframe than the current review. Their analysis indicated significant associations of the -403G/A and -28C/G polymorphisms in the *RANTES/CCL5* gene with protection against HIV-1 infection. Furthermore, their work suggested that the In1.1T/C polymorphism may increase susceptibility to HIV-1 infection, and such findings were corroborated in the current updated meta-analysis. Additionally, separate meta-analyses focused on two of these SNPs—rs2107538 and rs2280789—reinforced the associations found in this review. For the rs2107538 SNP, the data suggested a protective role against HIV-1 infection among African and Asian populations. For the rs2280789 SNP, resistance to infection was observed in Asian populations. These results align with the data reported in the present review [39,50,51].

Through a variety of methods, including gene therapy for immunomodulation, the target gene can be analyzed for potential therapeutic uses in HIV-1-infected individuals, such as those already heavily investigated for its receptor, CCR5 [52]. As a result, there are numerous attempts being made globally to develop RANTES compounds with strong anti-HIV-1 activity for use as microbicides or CCR5 antagonists without activating CCR5 [53,54]. In addition, when infected with the macrophage- and T-cell-tropic R5 and X4 HIV-1 viral strains, respectively, the chemokine receptors CCR5 and CXCR4 are crucial coreceptors for viral entrance. As a result, they make good siRNA-mediated downregulation targets. When creating efficient treatments, it is crucial to take into account blockage of each respective coreceptor because both the R5 and X4 viral types are engaged in disease pathogenesis [55].

Moreover, the ability to treat children who contracted HIV through mother-to-child transmission in the previous ten years as well as new cases of adolescents infected by HIV through other transmission routes has also been made possible by advancements in diagnostic and therapeutic technologies, which have reduced child and adolescent death rates and morbidity [56]. Thus, the findings of this present meta-analysis might aid in developing a scientific foundation for efficient HIV/AIDS control measures.

Further research is indeed warranted to validate the findings of this meta-analysis. Future genomic and epidemiological studies, including case-control, cohort, and cross-sectional designs, should consider larger sample sizes and more diverse ethnic and national backgrounds. These studies could help elucidate the genetic mechanisms that influence the human body’s molecular machinery. Understanding these mechanisms may pave the way for new therapeutic approaches that target specific chemokine sites or receptors or even serve as predictive markers for individuals infected with HIV-1.

The current work offers a hypothesis concerning the functional relationship of the identified SNPs to the risk of AIDS, particularly through the lens of the CCR5-CCL5 immune axis. Based on the data evaluated, the presence of mutant alleles in the analyzed SNPs—which are associated with increased expression of *CCL5/RANTES*—seems to confer resistance to AIDS. Conversely, SNPs linked to decreased protein levels of CCL5/RANTES may be associated with increased susceptibility to infection. This could potentially be related to induced hyperinflammatory responses. Therefore, these findings offer valuable insights into how SNPs in the CCR5-CCL5 immune axis might modulate susceptibility to HIV-1 infection and disease progression [57].

The following are some of the study’s limitations: (1) the unique definition of HIV-1 infection used by each study based on case identification; (2) the exclusion of studies involving the *CCR5* gene because it is a different gene and produces different polymorphisms; (3) the heterogeneity of SNPs acting as a potential bias in characteristics like ethnicities and ages of different populations due to the genetic background phenomenon; and finally, the study’s inability to account for all studies that were conducted; (4) only SNPs that were referred to in the National Center for Biotechnology Information (NCBI) were included; (5) the requirement for data analysis in investigations of various HIV-1 variations; (6) potential analyses of additional *RANTES/CCL5* genetic polymorphisms; (7) the subtype of HIV-1 in cohort individuals investigated in each study included; and (8) the employed search methodology.

## 5. Conclusions

This meta-analysis provides an overview of host immunogenetic factors of *CCL5/RANTES* associated with the HIV-1 disease risk. The SNPs rs2107538 and rs2280789, related to the higher expression of the *CCL5/RANTES* gene, were significantly associated with resistance to infection in several populations. Our summarized data indicate that SNPs associated with the function of decreasing its protein levels are related to AIDS susceptibility. Yet, further studies are needed for a definite conclusion. Finally, more genetic studies on HIV-1 may reveal new paths for therapeutic approaches, in addition to analyzing the influence of individual genetic characteristics on the host’s immune response in this context of infection.

## Figures and Tables

**Table 1:** Characteristics of the studies included in this review.

N°	Authors and Year of Publication/Database/JBI Score	Title	Country or Region/Ethnicity	Type of Study/Participants	Applied Molecular Biology Method/Source of Control	HWE Testing (*p*-Value)	SNP ID (Mutation)	Association
1	(LIU et al., 1999a) [14]/PUBMED/(10/11)	Polymorphism in RANTES chemokine promoter affects HIV-1 disease progression	Japan/Asian	Cohort study/272 cases and 193 controls.	PCR-SDCT/Population-based	*p* > 0.05	rs2107538; rs2280788.	For the SNP rs2107538, no significant associations were observed. For the SNP rs2280788, the presence of the mutant allele (G): less susceptibility.
2	(LIU et al., 1999b) [11]/PUBMED/(11/11)	Distribution of HIV-l disease modifying regulated on activation normal T cell expressed and secreted haplotypes in Asian, African and Caucasian individuals	Ivory Coast, Japan, China, France, Thailand/Asian and European	Cohort study/221 cases and 353 controls.	PCR-RFLP/Population-based	*p* > 0.05	rs2107538; rs2280788.	For the SNP rs2107538, the presence of the mutant allele (A): less susceptibility. For the SNP rs2280788, the presence of the mutant allele (G): higher risk.
3	(MCDERMOTT et al., 2000) [23]/PUBMED and MEDLINE/(11/11)	Chemokine RANTES promoter polymorphism affects risk of both HIV infection and disease progression in the Multicenter AIDS Cohort Study	West Africa and USA–USA/North American White, Hispanic, and Black, Asians, and Africans.	Cohort/123 exposed-uninfected individuals and 506 HIV-positive subjects.	PCR-RFLP/Population-based	*p* > 0.05	rs2107538; rs2280788.	Both SNPs (presence of mutant allele): higher risk of HIV-1.
4	(GONZALEZ et al., 2001) [24]/PUBMED/(11/11)	Global survey of genetic variation in CCR5, RANTES, and MIP-1α: Impact on the epidemiology of the HIV-1 pandemic.	USA/European-, African-, and Hispanic-American	Cohort/506 cases and 123 controls.	PCR-SDCT/Population-based	*p* > 0.05	rs2107538; rs2280788.	No association for these SNPs.
5	(FERNÁNDEZ et al., 2004) [25]/PUBMED/(11/11)	Fluorescence Resonance Energy Transfer Analysis of the RANTES Polymorphisms -403G → A and -28G → C: Evaluation of Both Variants as Susceptibility Factors to HIV Type 1 Infection in the Spanish Population.	Spain/European	Cohort/440 cases and 100 controls.	PCR-SDCT/Population-based	*p* > 0.05	rs2107538; rs2280788.	Lack of association between these SNPs and HIV-1 infection.
6	(SURESH et al., 2006) [26]/PUBMED/(10/11)	Gene polymorphisms in CCR5, CCR2, CX3CR1, SDF-1 and RANTES in exposed but uninfected partners of HIV-1 infected individuals in North India	India/Asian	Cohort/110 cases and 50 controls.	PCR-RFLP/Hospital-based	*p* > 0.05	rs2107538; rs2280788.	No association was observed for these SNPs.
7	(WANG et al., 2004) [27]/PUBMED/(11/11)	Cytokine and chemokine gene polymorphisms among ethnically diverse North Americans with HIV-1 infection	USA/African-American and Hispanic-American	Cohort/319 cases and 258 controls.	PCR-SSPs/Population-based	*p* > 0.05	rs2107538; rs2280789.	SNP rs2107538 (presence of allele A) was associated with higher susceptibility to HIV-1 infection. No association for SNP rs2280789 in this cohort.
8	(ZHAO et al., 2004) [28]/PUBMED	Effects of single nucleotide polymorphisms in the RANTES promoter region in healthy and HIV-infected indigenous Chinese	China/Asian	Cohort/249 cases and 1028 controls.	PCR-RFLP/Hospital-based	*p* > 0.05	rs2107538; rs2280788.	No association.
9	(AHLENSTIEL et al., 2005) [29]/PUBMED/(8/8)	Distribution and effects of polymorphic RANTES gene alleles in HIV/HCV coinfection–A prospective cross-sectional study	Germany/European	Cross-sectional study/85 cases and 109 controls.	PCR-FRET/Hospital-based	*p* > 0.05	rs2107538; rs2280788; rs2280789.	All these SNPs (presence of the variant allele) showed an association with higher risk for this infection.
10	(WICHUKCHINDA et al., 2006) [30]/PUBMED/(10/11)	Protective effects of IL4-589T and RANTES-28G on HIV-1 disease progression in infected Thai females	Thailand/Asian	Cohort/246 cases and 119 controls.	PCR-RFLP/Hospital-based	*p* > 0.05	rs2107538; rs2280788; rs2280789.	No association for these SNPs.
11	(VIDAL et al., 2006) [31]/PUBMED/(10/10)	Polymorphism of RANTES chemokine gene promoter is not associated with long-term nonprogressive HIV-1 infection of more than 16 years	Spain/European	Case-control/167 cases and 100 controls.	PCR-SDCT/Population-based	*p* > 0.05	rs2107538; rs2280788.	No association for these SNPs.
12	(RATHORE et al., 2008) [32]/PUBMED/(10/11)	Association of RANTES -403 G/A, -28 C/G and In1.1 T/C polymorphism with HIV-1 transmission and progression among North Indians	India/Asian	Cohort/196 patients and 362 controls.	PCR-RFLP/Population-based	*p* > 0.05	rs2107538; rs2280788; rs2280789.	Only the SNP rs2280789 showed a significant association, the mutant allele C: protection from the disease.
13	(LI et al., 2014) [33]/PUBMED/(11/11)	Gene polymorphisms in CCR5, CCR2, SDF1 and RANTES among Chinese Han population with HIV-1 infection	China/Han Chinese	Cohort/287 cases and 437 controls.	PCR-RFLP/Population-based	*p* > 0.05	rs2107538; rs2280788.	Only the SNP rs2107538 showed a significant association, the mutant allele A: protection from the disease.
14	(MHANDIRE et al., 2014) [34]/PUBMED/(10/11)	CCR2, CX3CR1, RANTES and SDF1 genetic polymorphisms influence HIV infection in a Zimbabwean pediatric population	Zimbabwe/African	Cohort/33 cases and 36 controls.	PCR-SNaPshot/RFLP/Population-based	*p* > 0.05	rs2107538; rs2280789.	No association for these SNPs.
15	(CELERINO DA SILVA et al., 2016) [35]/PUBMED and SciELO/(11/11)	Chemokines SNPs in HIV-1+ Patients and Healthy Controls from Northeast Brazil: Association with Protection against HIV-1 Infection	Brazil/Not reported	Cohort/286 cases and 221 controls.	PCR Genotyping/Population-based	*p* > 0.05	rs2280789.	No significant association.
16	(MUTUIRI et al., 2016) [36]/PUBMED/(10/11)	RANTES Gene Polymorphisms Associated with HIV-1 Infections in Kenyan Population	Kenya/African	Cohort/100 cases and 100 controls.	PCR-RFLP/Hospital-based	*p* > 0.05	rs2107538; rs2280788.	No association for these SNPs.
17	(VEGA et al., 2017) [37]/PUBMED/(11/11)	Haplotypes in CCR5-CCR2, CCL3 and CCL5 are associated with natural resistance to HIV-1 infection in a Colombian cohort	Colombia/Latin American	Cohort/19 patients and 14 controls.	PCR-RFLP/Hospital-based	*p* > 0.05	rs2107538; rs2280788.	Lack of association for these SNPs.

## Data Availability

The original contributions presented in the study are included in the article. Further inquiries can be directed to the corresponding author.
